# Supplementary material for: The auditory P2 is influenced by pitch changes but not pitch strength and consists of two separate subcomponents
Source: Imaging Neurosci (Camb). 2024 May 9;2:imag-2-00160. doi: 10.1162/imag_a_00160 (PMC12247569; doi:10.1162/imag_a_00160)
Supplement: Supplementary Material [file imag_a_00160-supp.pdf]

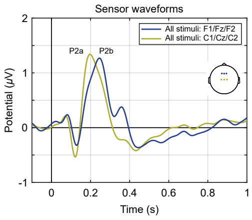

Supplemental Figure 1. Sensor-level ERPs. ERP traces, averaged across all stimulus conditions, for three electrodes in the central (C1, Cz, C2) and fronto-central (F1, Fz, F2) scalp regions. At central sites, the P2 peaked notably earlier ("P2a") than at fronto-central sites ("P2b").
